# Return to Work in Patients with Chronic Musculoskeletal Pain: Multidisciplinary Intervention Versus Brief Intervention: A Randomized Clinical Trial

**DOI:** 10.1007/s10926-016-9634-5

**Published:** 2016-02-24

**Authors:** Randi Brendbekken, Hege R. Eriksen, Astrid Grasdal, Anette Harris, Eli M. Hagen, Tone Tangen

**Affiliations:** 1 Department of Physical Medicine and Rehabilitation, Innlandet Hospital Trust, Ottestad, Norway; 2 Department of Sport and Physical Activity, Bergen University College, Uni Research Health, Bergen, Norway; 3 Department of Economics, University of Bergen, Bergen, Norway; 4 Department of Psychosocial Science, Faculty of Psychology, University of Bergen, Bergen, Norway; 5 Department of Clinical Medicine, Haukeland University Hospital, University of Bergen, Bergen, Norway

**Keywords:** Return to work, Sick leave, Chronic pain, Work disability, Coping

## Abstract

*Objective:* This randomized clinical trial was performed to compare the effect of a new multidisciplinary intervention (MI) programme to a brief intervention (BI) programme on return to work (RTW), fully and partly, at a 12-month and 24-month follow-up in patients on long-term sick leave due to musculoskeletal pain. *Methods:* Patients (n = 284, mean age 41.3 years, 53.9 % women) who were sick-listed with musculoskeletal pain and referred to a specialist clinic in physical rehabilitation were randomized to MI (n = 141) or BI (n = 143). The MI included the use of a visual educational tool, which facilitated patient-therapist communication and self-management. The MI also applied one more profession, more therapist time and a comprehensive focus on the psychosocial factors, particularly the working conditions, compared to a BI. The main features of the latter are a thorough medical, educational examination, a brief cognitive assessment based on the non-injury model, and a recommendation to return to normal activity as soon as possible. *Results:* The number of patients with full-time RTW developed similarly in the two groups. The patients receiving MI had a higher probability to partly RTW during the first 7  months of the follow-up compared to the BI-group. *Conclusions*: There were no differences between the groups on full-time RTW during the 24 months. However, the results indicate that MI hastens the return to work process in long-term sick leave through the increased use of partial sick leave.

*Trial Registration*: http://www.clinicaltrials.gov with the registration number NCT01346423.

## Introduction

Musculoskeletal disorders are amongst the primary causes of work disability in Western societies and thereby represent enormous costs to the community in economic terms [1]. Painful disorders of the back, neck and upper limbs are the most frequently used diagnoses, with sickness absence, long-term incapacity for work and permanent disability as frequent consequences [2]. In Norway nearly half of all sickness absence is due to a musculoskeletal pain diagnosis with low back pain (LBP) as the largest single cause [3].

Health measures in Western societies are improving, but sickness benefits and disability claims due to musculoskeletal disorders increase [4]. Maintaining activity including work, in spite of muscular pain, is an important part of the recovery process as the opposite delays recovery [5–7]. The process of return to work (RTW) is therefore clearly a major concern in this patient group [8, 9].

The journey from acute muscle pain to long-term sickness, work absenteeism and disability has been widely investigated. Such studies have revealed that psychological and social factors, as well as somatic pathology, influence chronicity and disability [10]. When the duration of sickness-absence due to musculoskeletal pain exceeds 8 weeks, the prognosis worsens and the probability of RTW is reduced [8, 9, 11].

The process of RTW in chronic pain can be conceptualized as a complex human behaviour change, where the patient her/himself takes the final decision on RTW or not. However, the general practitioner (GP) is the main gatekeeper of access to sickness benefits [12]. The patient’s own evaluation of their RTW is influenced by several personal, social, economic and work-related factors [13–15]. According to behaviour models, a change in behaviour is influenced by knowledge, attitudes, norms and self-efficacy [15, 16]. Banduras [17] Social Cognitive Theory posits a multifaceted causal structure to explain how human motivation, behaviour and well-being are regulated. In this model, self-efficacy beliefs, goals, outcome expectations and perceived environmental impediments and facilitators, all operate together as regulators of motivation and behaviour. This corresponds to the suggestion that interventions in sick-listed, chronic pain patients should not primarily focus on pathology but rather, on adapting to a complex situation which should include giving more attention to coping, self-management skills, environmental factors, workplace support and patient education [18]. This may enhance the patient’s positive response outcome expectancies (coping). According to the Cognitive Activation Theory of Stress (CATS), such improvements will dampen the stress response, which, in the next step, might help patients towards a more constructive handling of complaints [19].

In general, the multidisciplinary approach (MDA) is accepted as a reasonable approach to treat chronic pain patients, as this should be regarded as multicausal [20–22]. In a recent Cochrane review, MDA was found to be favourable in decreasing pain and disability compared to usual care [23]. However, the effects on the RTW of multidisciplinary interventions for chronic muscular pain have been conflicting [7, 23, 24].

The majority of chronic musculoskeletal pain conditions including LBP, are characterized by the lack of objective, pathological findings although the patients present numerous additional subjective health complaints and experience reduced work ability [25]. The GP’s assessment concerning sick leave must, to a great extent, rely on the patient’s description of his/her condition in combination with the GP making an effort to understand the workplace environment and the actual work demands. Several studies have revealed a need to expand clinicians practice in this field, as many GP’s do not readily engage in workplace discussions with the patient [26, 27]. There is growing evidence that occupational factors influence disability and that GP’s proactive communication related to health and workplace strategies is of major importance to RTW [28]. This calls for approaches where clinicians more actively assess occupational factors and health complaints together in the rehabilitation process.

In this study, we applied a multidisciplinary intervention (MI) that is tailored to highlight the complexity of long-term pain problems. The MI included an assessment of work, family situation, lifestyle, coping strategies and health problems. The MI applied a novel educational tool, the Interdisciplinary Structured Interview and a Visual Educational Tool (ISIVET), to establish an overall picture of the patient’s situation through visualization. The underlying hypothesis was that this design could introduce a new cognitive approach to cope with health problems. This might strengthen the motivation of patients to go through with changes, thereby improving the actual coping and resuming work. The active control group received a brief intervention programme (BI), based on a non-injury model which has proved particularly effective on RTW in patients with sub-acute LBP [29–32]. The non-injury model is based on the understanding of the back or the body as a robust structure where pain should not necessarily be taken as a sign of injury caused by inappropriate behavior or any wrongdoing. This view is communicated to reduce pain-initiated fear and secondly to encourage natural movements and reduce tense and awkward movements which often come from the belief that pain is caused by an injury of the body and that care, protection and restrictions are mandatory which comply with the injury-model.

### Objectives

The objective of the study was to test if a MI is more effective than a BI on RTW in patients sick-listed due to musculoskeletal pain. We hypothesized that the MI would be superior to BI in increasing RTW over a period of a 24-month follow-up.

## Materials and Methods

### Study Design, Recruitment and Participants

This study was a randomized clinical trial which took place at two different outpatient clinics at the Department of Physical Medicine and Rehabilitation (DPMR), Innlandet Hospital Trust, Norway, from 2011 to 2013. All of the patients from two different counties in the south–eastern part of Norway, sick-listed for musculoskeletal pain and referred to the DPMR, were considered for participation. The study followed the CONSORT statement for reporting of randomized trials.

The inclusion criteria were: aged between 20 and 60 years, a sick leave degree between 50 and 100 % due to musculoskeletal pain and for <12 months, and at least 50 % employment contract. The exclusion criteria were: pregnancy, current cancer, osteoporosis, recent physical trauma/injury, serious mental illness, rheumatic inflammatory diseases, not capable of understanding and speaking Norwegian, or being involved in an on-going health insurance claim.

**Fig. 1:**
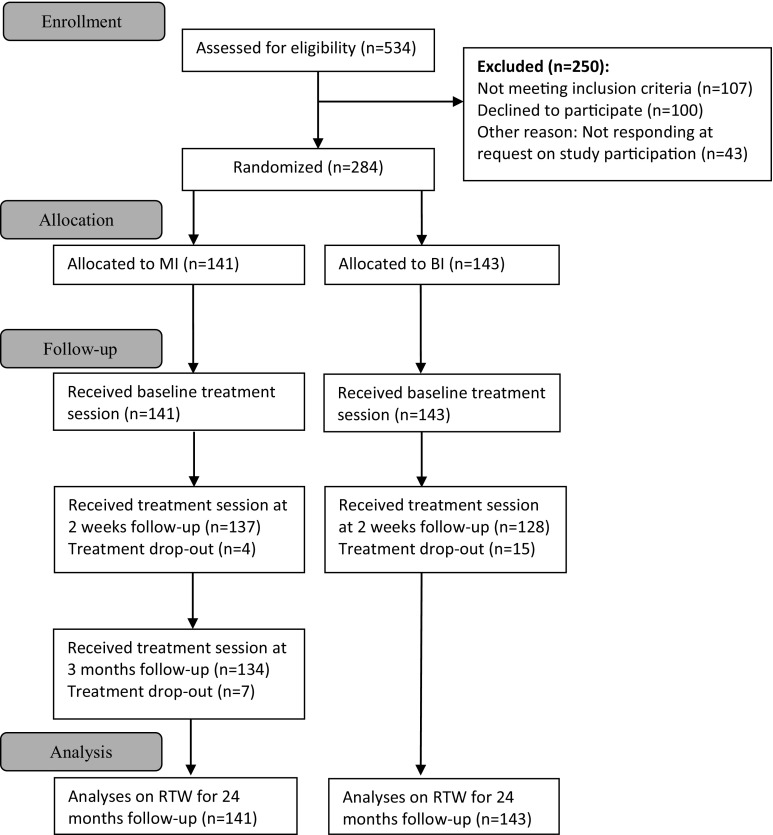
Flowchart of participation in treatment sessions: *MI* multidisciplinary intervention, *BI* brief intervention, *RTW* return to work

A total of 534 patients were screened for eligibility, whereby 250 were found to not be eligible for different reasons (Fig. 1). This study included 284 patients referred from 136 different GPs. These patients were randomized to either MI (n = 141) or BI (n = 143). The two interventions were performed by different teams and no clinician working in the MI-team ever worked in the BI-team. The time from inclusion/randomization to baseline assessment at the clinics was between one and 2 weeks.

### Context

All lawful residents of Norway are included in the Norwegian public insurance system. This provides health service benefits and pensions for all members of the National Insurance Scheme, administered by the Norwegian Welfare and Labour Administration (NAV). When a worker, due to a medically acknowledged disease, is sick-listed by his/her GP, the workers’ compensation programme, which is administered by NAV, provides 100 % coverage for lost income from day one until the person can work again, up to 52 weeks. The employer covers the first 16 days. After 1 year, the NAV covers the long-term rehabilitation benefits or disability pension, providing approximately 66 % of the patient’s former income. These benefits can also be combined with work if the disability constitutes a minimum of 50 %.

### Interventions

#### The Multidisciplinary Intervention with the ISIVET

##### Baseline Assessment

**Fig. 2:**
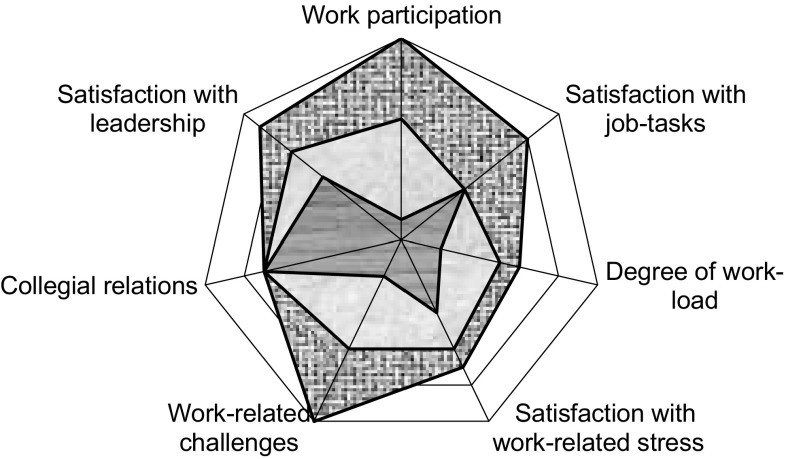
The ISIVET-figure “Working conditions”, assessed three times

Initially, the patient met each of the three members of the multidisciplinary team successively (social worker, physician and physiotherapist). The social worker first interviewed the patient about their family life, social life, education and economics and then collaborated with the patient on scoring the ISIVET-figure “Working conditions” (Fig. 2). This evaluated seven different issues: work-related stress, satisfaction with job-tasks, workload, collegial relationships, leadership, degree of challenges at work and occupational participation.

The physician first interviewed the patient about the family’s health, as well as his/her former and present health. Then the physician conducted a physical examination, concluding with an ICD-10 diagnosis. Finally the physician and the patient collaborated on scoring the ISIVET-figure “Quality of life”. This evaluates the following issues: physical complaints, psychological wellbeing, sleep, energy, physical activity, social participation and occupational participation.

The physiotherapist assessed the musculoskeletal problems of the patient and conducted a physical examination.

The ISIVET comprised a manual and the two figures, which were star-plots with seven axis representing seven different issues. The scores on each axis were set between 1 and 10, with “10” positioned in the periphery of the star-plot, indicating an optimally positive situation. Meanwhile, “1” was located in the centre of the star-plot, indicating a maximum negative situation. The manual gave illustrating examples of situations at different scoring levels and, through discussion, the patient and the clinician identified the right score for every issue. When all of the scores were completed, a line was drawn between the scoring points, which produced an area in each figure. The area was coloured for better visualization for the patient, as well as for the clinicians. The problem areas were demonstrated by a lack of colour, while existing resources stood out as the coloured area.

When the sessions with the three clinicians were completed, the whole team met briefly to share their findings and general impression of the patient and her/his situation. The possible barriers to work-participation, maintaining factors to the pain problem and eventually other important issues, were highlighted. Following this, the patient joined the team for an evaluation on the total situation including health problems and work. The two figures with their coloured areas were central in this phase and when discussing problem solving and possible fields of actions. The patient played a major role in deciding the ways to go forward, with guiding from the areas and with the team as a counselling partner. The agreement on the actions was written down in a list, which constituted the patient’s rehabilitation plan. The actions were typically related to the handling of pain and fear avoidance, to lifestyle, particularly physical activity, and to family or work matters. When leaving the clinic, the patient received a paper-copy of the ISIVET-figures with the coloured areas and the rehabilitation plan listed as the points to be followed. The complete baseline assessment lasted 3.5 h.

##### Two-Week Follow-Up

The patient met the physiotherapist for 1 h to evaluate the rehabilitation plan and work through the ISIVET once more. New areas were coloured with a second colour (Fig. 2). The visualization of the delta-areas was a matter of attention and reflection. Previous advice and actions were highlighted according to this, and adjustments to the rehabilitation plan were eventually made.

##### Three-Month Follow-Up

The patient and the whole team met for 1 h to sum up the situation and evaluate the interventions so far. The ISIVET was worked through and new areas were coloured with a third colour. Eventually, they adjusted the rehabilitation plan.

During the study period, four different physicians, all specializing in physical medicine and rehabilitation, two different social workers, and four different physiotherapists were engaged in the MI-treatment. The total face-to-face-time spent with the patient during the MI was 5.5 h.

#### The Brief Intervention (BI)

BI as applied in this study, is based on the studies by Indahl [30, 31] and Hagen [29], and we used the modified version of BI which is described in Hagens work.

The BI comprised two sessions: a baseline session lasting about 2.5 h including separate consultations with a physician and a physiotherapist, and a two-week follow-up with the physiotherapist for about 1 h.

The BI is based on a non-injury-model for LBP. It aims to reduce fear and concern and help the patient to stay active despite the pain, unless “red flags” [33] are identified, emphasizing the fact that the back is a strong and robust structure and that return to normal activity would be beneficial. The essential feature of the method is giving the patient time to express problems, worries and thoughts. This is followed by a thorough medical, educational examination, where any somatic findings are explained to the patient. The patient is informed about the good prognosis and importance of staying active.

Therapist treatment manuals were based on the current guidelines [7] and the manual used by Hagen [29]. A physician, who was a specialist in physical medicine and rehabilitation, and a physiotherapist, carried out the BI. Both of the therapists were experienced in the method. The total face-to-face-time spent with the patient during the BI was 3.5 h.

### Data and Outcome

The social insurance register provides information about the start and stop dates for payments of sickness benefit, rehabilitation benefits, disability pension and unemployment benefits. For payments of sickness benefits and disability pension, we have information about the degree of disability and hence, indirectly, the degree of work participation. Only the payments for absences exceeding 16 days are refunded by the National Insurance. Therefore, absences that last 16 days or less are not included in our data.

We used the register data to define the work/social insurance status in each calendar month after inclusion in the trial. The register data provided follow-up data on every participant in both treatment groups for the 24-month follow-up.

Due to the inclusion criteria, all of the participants were employed and on sickness benefits at baseline. We defined that, if more than 50 % of the working days in a given calendar month were spent on full-time sick leave, the status for that month was given as “out of work” (OOW). If more than 50 % of the working days in a given calendar month were spent on partly sick leave, the status for that month was given as “partly return to work” (p-RTW). If no benefits were provided in more than 50 % of the working days, the status for that month was “fully return to work” (f-RTW). From these data, we constructed a file where each study participant had one of three possible statuses each month for the 24-month follow-up: OOW, p-RTW or f-RTW.

The primary outcome of this study was RTW fully and partly, at the 12-month and 24-month follow-up.

### Sample Size

The sample size calculations were based on the results from a previous RCT on BI in low-back pain [29]. With a power of 80 % and a significant level of 5 %, the total number of participants needed for this study was calculated to be 300.

### Randomization and Blinding

The randomization was concealed and the patients were randomized to either MI or BI, according to a computer-generated randomization-list, which was set up by a statistician at Uni Research Health (URH). The list was stratified by age and gender. A research assistant, who was not involved in the treatment, contacted URH and was informed about which treatment the patient should receive. There was no blinding to the treatment of therapists or participants.

### Statistical Methods

**Fig. 3:**
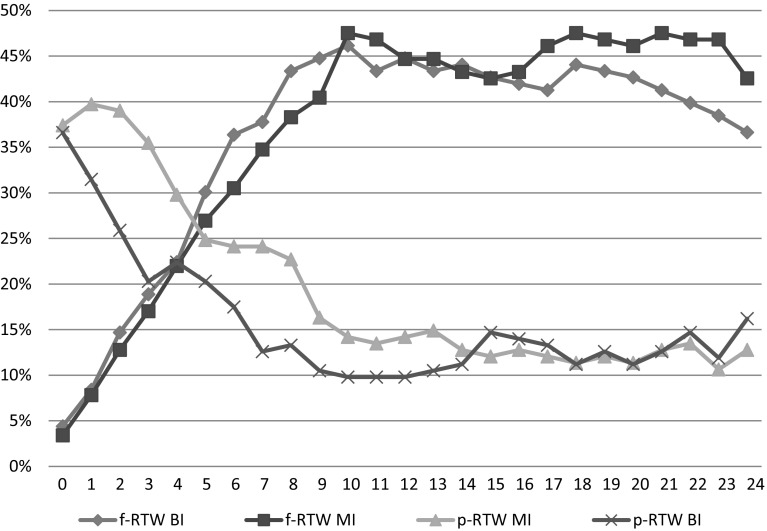
Descriptive statistics on work status in valid % of multidisciplinary intervention group (MI) and brief intervention group (BI): proportions fully returned to work (f-RTW), partly returned to work (p-RTW) for both groups at each month for 24 months follow-up

Descriptive statistics based on the groups for the 24-month follow-up were performed (Fig. 3), in addition to a multinomial logistic analysis to explore the relative risk (RR) ratios for p-RTW and f-RTW between the groups every month (Table 2). *P* values <0.05 was considered statistically significant. The analyses adhered to the “intention-to-treat” principle. The data were analysed using SPSS 21.

### Ethical Considerations

The research was carried out in compliance with the principles in the Helsinki declaration. The Norwegian Regional Ethical Committee and the Norwegian social science data services approved the study [34, 35]. Personal confidentiality was guaranteed and informed consent was signed by each participant, with emphasis on the right to withdraw from the study at any time without any explanation.

## Results

### Patient Characteristics

The baseline characteristics of the patients are presented in Table 1. The mean duration of sick leave by inclusion was 147 days (SD = 60.1). Due to the waiting time from inclusion to baseline assessment (between one and 2 weeks), 11 patients (3.9 %) were no longer sick-listed by baseline. Education limited up to 12 years was more predominant in the MI-group (73.8 %) compared to the BI-group (63.3 %). The dominant diagnoses in accordance with ICPC-2 [36] were: low back pain L02/L03/L84/L86 (39.5 %), neck pain L01/L83 (12.1 %), widespread pain/fibromyalgia L18 (10.7 %) and shoulder pain L08/L92 (7.8 %). The study-population was given a total of 51 different diagnoses. Of these, the L-group represented 84.2 %. There were no significant differences between the intervention groups regarding sick-leave duration or distribution of the different medical conditions by baseline.

**Table 1:** Demographic and clinical characteristics by baseline [number (n) and valid percent (%)], divided by intervention groups, multidisciplinary intervention (MI) and brief intervention (BI)

Variables	MI (n = 141) n (%)	BI (n = 143) n (%)
Demographic		
Age (mean, SD)	40.9 (9.8)	41.6 (9.5)
Women	77 (54.6)	76 (53.1)
Married or cohabitant	107 (75.9)	110 (77.0)
Children		
None	25 (17.7)	31 (21.7)
1–2	75 (53.2)	73 (52.5)
Level of education		
Public school, 1–12 years	104 (73.8)	91 (63.6)
University/college, >12 years	24 (17.0)	28 (20.6)
Work related variables		
Employment degree		
Partial (≥50 %, <100 %)	39 (28.3)	30 (25.4)
Full time	99 (71.7)	103 (74.6)
Working time		
Shift^a^	47 (34.6)	52 (38.2)
Sick-leave degree		
Partial (≥50 %, <100 %)	51 (36.2)	52 (36.4)
Full-time	85 (60.4)	85 (59.2)
Job security: *“Do you have a job to return to?"*		
Yes	124 (91.9)	127 (92.0)
Demands at work		
Physically demanding	76 (55.1)	74 (52.5)
Mentally demanding	40 (29.2)	28 (19.9)

^a^Working both day and night-time

### Return to Work

There were no differences between the MI-group and BI-group on f-RTW during the follow-up period of 24 months. The highest RR was 1.42 (95 % CI 0.87–2.33, *p* = 0.17), which was in the 23rd month. In all of the other months, the RR was closer to unity (Table 2, Fig. 3).

**Table 2:** Partly return to work (p-RTW) and fully return to work (f-RTW) for the Multidisciplinary Intervention group compared to the Brief Intervention group (reference group)

Months follow-up	p-RTW		f-RTW	
	RR	95 % CI^a^	RR	95 % CI^a^
1	1.45	0.88–2.39	1.07	0.44–2.56
2	1.86	1.10–3.14*	1.07	0.53–2.18
3	2.24	1.28–3.91**	1.15	0.61–2.18
4	1.53	0.87–2.68	1.13	0.62–2.03
5	1.26	0.70–2.28	0.92	0.53–1.60
6	1.40	0.75–2.61	0.85	0.50–1.45
7	2.31	1.19–4.51*	1.11	0.66–1.87
8	1.90	0.97–3.72	0.98	0.59–1.64
9	1.61	0.77–3.37	0.93	0.57–1.54
10	1.67	0.77–3.61	1.18	0.72–1.95
11	1.62	0.75–3.53	1.27	0.78–2.09
12	1.60	0.74–3.46	1.10	0.67–1.81
13–23	Results not reported			
24	0.85	0.42–1.71	1.25	0.75–2.06

Differences between the groups were estimated by multinomial regression analysis with “fully out of work” as reference category. Risk Ratio (RR), 95 % Confidence Interval (CI) with *p* values are presented

^a^Indicates *p* value

* *p* value <0.05; ** *p* value <0.01

At 12 months of the follow-up, 63 patients (44.7 %) in the MI-group and 64 patients (44.8 %) in the BI-group were f-RTW. The corresponding numbers at 24 months were: 60 (42.6 %) in the MI-group and 52 (36.6 %) in the BI-group.

In three of the first 7 months after baseline, significantly more patients were p-RTW in the MI-group compared to the BI-group. The highest RR was at the seventh month: RR = 2.31 (95 % CI 1.19–4.51, *p* = 0.01). The corresponding numbers for the second month was: RR = 1.86 (95 % CI 1.10–3.14, *p* = 0.02) and for the third month: RR = 2.24 (95 % CI 1.28–3.91, *p* < 0.01).

By 12 months, 59 patients (41.8 %) in the MI-group and 65 patients (45.5 %) in the BI-group were still OOW. The corresponding numbers by 24 months were 63 (44.7 %) in the MI-group and 68 (47.6 %) in the BI-group.

## Discussion

In this study of patients on long-term sick leave due to musculoskeletal pain, there were no significant differences on RTW between the patients who received MI or the patients receiving BI at 12 months or 24 months of the follow-up. However, patients in the MI-group returned to work faster than patients in the BI-group. This is illustrated by the differences between the groups in proportions fully out of work (OOW): At 3 months of the follow-up, the proportion OOW in the MI-group was reduced to 48 %, while in the BI-group it was slightly increased to 61 % compared to the baseline levels.

A number of factors prolong musculoskeletal pain. Some are obviously related to the individual, others to the workplace [37] or to compensation systems [38]. Multidisciplinary interventions comply with the possibility that barriers to work-participation exist at multiple levels and have proven beneficial to facilitate RTW in low back pain [23]. As psychosocial factors predict the long-term incapacity of musculoskeletal disorders [39], interventions focusing on these aspects should be of clinical value.

In our study, the MI-group received a more comprehensive approach, which included more therapist time, one more profession, more focus on psychosocial factors, in particular work and workplace adaptions, compared to the BI-group. The 2 h difference in therapist time might contribute to improved results on p-RTW in the MI-group. The MI also applied the ISIVET, which was constructed to improve patient-therapist communication, facilitate patient and therapist insight in the entirety of the situation, and improve the patient’s autonomy and thereby, their engagement in their own rehabilitation. The ISIVET aimed to raise awareness of the value of work participation through visualization of large areas in the star plot. It also aimed to motivate patients to choose to work at least partially, if not fully, in spite of their health complaints, with the suggestion that work is healthy. If areas were small, the possibility of alternative work was questioned by the clinicians.

However, the MI did not increase the proportion of patients who were f-RTW at 12 months or 24 months, compared to the BI. However, the results of p-RTW are in accordance with the conclusion of a recent Cochrane review and of the findings of Loisel and his co-workers, where a combination of a clinical intervention and an occupational intervention was associated with a faster RTW [40, 41]. The workplace intervention in the MI-group was limited to the discussion and planning of workplace adaptions between the patient and the team at the clinic. The patient was responsible for initiatives at the workplace, which was part of the patient-oriented coping strategy for the MI.

The MI-group improved faster than the BI-group on mental and physical symptoms, functional ability and coping, but these results are published elsewhere [42].

The treatment of musculoskeletal pain is primarily given by the GP but more complex cases are eventually referred to the specialist health care [7, 20]. In this study, the GPs who referred the patients did not know that their patients might be enrolled in a clinical study. It is reasonable to assume that our study-population is regarded by the GPs as difficult to treat, as they were referred to specialist health care and on long-time sick leave with musculoskeletal pain, which in itself gives a poor prognosis. This might explain the relatively low RTW-proportion in both groups. It might also explain why it was difficult to achieve better results, even with a more comprehensive approach and in spite of improvements in health, coping and function abilities, as described in an earlier paper [42].

A possibility that the patient is determined not to go back to work or, for some reason, do not want to return to their former workplace represents information that is not necessarily accessible to the therapists. The majority of the participants had low education and physically demanding jobs, thereby representing mostly blue-collar workers with fewer opportunities to find alternative work. This might also contribute to the low proportions of RTW in this study.

The Norwegian sickness compensation system offers 100 % salary compensation from day one for up to a year. After that period, the patient is covered by 66 % compensation of salary through a rehabilitation allowance or disability pension. This generous compensation system might undermine the process of RTW through weak economic incentives for the patients to get out of sick leave in both groups.

The MI-group had a total of three sessions with therapists during a three-month period and the BI-group had two sessions. Given that these patients were on long-time sick leave, it was perhaps too optimistic to hypothesize that a limited intervention would increase RTW extensively.

### Limitations and Strengths of the Study

The primary strengths of this study constitute the study design with the randomization giving comparable groups, and the relatively large sample included. Secondly, the use of register data, leaving us with information on work participation and sickness-compensation every month for 24 months of the follow-up for all of the patients included. Furthermore, both treatments were based on written manuals and were easy to describe. Different teams did the BI and the MI, reducing the possibility of mixing the two methods. The BI-method was well known to the therapists involved, and they had recently been videotaped and quality assured in performing BI in another trial [43]. The therapists performing the new MI-method practised regular meetings and supervision to ensure adherence to the protocol and equal practice of the method. The sickness-certificates were prescribed by the GP’s and not by the physicians in the study, reducing the possibility of a biased prescription from therapists in the study. Finally; the drop out of treatment was low in both groups indicating that the treatment is feasible in clinical practice and that the results are reliable.

Some limitations should also be mentioned. First of all; there were many similarities in the two treatment methods and this could influence the possibility to come out with significant differences in the outcome. Both were based on a non-injury- and a bio-psychosocial model in pain assessment, and both practised patient education. Furthermore, both methods had an intervention limited to the individual level; the patient. We could therefore not explore the effect of environmental factors, nor could the therapists involve a third part directly, which might have been valuable in the process considering the significance of psychosocial factors in chronic pain [4]. The occupational intervention in MI was limited to the assessments of different aspects of the work-situation and the discussion with the patients on possible fields of action, while the follow-up on the eventual workplace interventions was the patient’s own responsibility. This might not be sufficient to achieve actual changes. External support with RTW-planning and process might have improved the results as there is evidence that workplace intervention improves time until first and lasting RTW among workers with musculoskeletal disorders [41]. In the BI-group, there were fewer and more experienced therapists compared to the MI-group. If the therapists in the MI had less confidence of their role due to less experience, it might have influenced the interaction with the patient and trough this, the outcomes, as there is evidence that what the doctor and other health professionals say and do has a powerful influence on outcomes [44]. There was no use of audiotaping to ensure adherence to the protocol in this study and there was no blinding of patients or therapists of practical reasons. The patients knew they would get one out of two possible interventions. Both were given in the specialist health care and both were comprehensive compared to ordinary services patients experience in the health care system. The lack of blinding of the patients might therefore be a limited weakness to this study. And finally, multiple analyses were performed increasing the risk of finding significant differences by coincidence. However, the results on p-RTW showed a continuous trend towards differences in the first 14 months. This trend supports the validity of the three significant *p* values.

### Concluding Remarks

A comprehensive MI focusing on work and psychosocial factors could not increase RTW at 12 months and 24 months in patients with chronic musculoskeletal pain, when compared to the effect of a less resource-demanding BI. However, the MI hastened the return to work process through the increased use of partial sick leave during the first months of the follow-up, compared to the BI. Longer treatments that more actively involve the workplace, combined with structural changes in sickness compensation and labour marked, might be necessary to decrease the proportion of patients on long-term sick leave for musculoskeletal pain.
